# Sulphated Zirconia as an Eco-Friendly Catalyst in Acylal Preparation under Solvent-Free Conditions, Acylal Deprotection Assisted by Microwaves, and the Synthesis of Anhydro-Dimers of *o*-Hydroxybenzaldehydes

**DOI:** 10.3390/molecules14104065

**Published:** 2009-10-12

**Authors:** Laura Nadxieli Palacios-Grijalva, Deysi Y. Cruz-González, Leticia Lomas-Romero, Eduardo González-Zamora, Gerardo Ulibarri, Guillermo E. Negrón-Silva

**Affiliations:** 1 Departamento de Ciencias Básicas, UAM-A, Av. San Pablo, No. 180. C.P. 02200, México, D.F., Mexico; E-Mails: lnpg@correo.azc.uam.mx (L.N.P.-G.); dycg@correo.azc.uam.mx (D.Y.C.-G.); 2 Departamento de Química, UAM-I, San Rafael Atlixco, No. 186. C.P. 09340, México, D.F., Mexico; E-Mails: llr@xanum.uam.mx (L.L.-R.); egz@xanum.uam.mx (E.G.-Z.); 3 Department of Chemistry & Biochemistry, Laurentian University, Sudbury, ON P3E2C6, Canada; E-Mail: gulibarri@laurentian.ca

**Keywords:** sulphated zirconia, solvent-free, microwaves, regioselective, diacetates

## Abstract

A solvent-free approach is described for the regioselective synthesis of acylals (1,1-diacetates) in shorter reaction times and higher yields, compared to conventional methodology using solvents. In the protection reaction of the *o*-hydroxybenzaldehyde the formation of acetyl compounds and anhydro-dimers was observed. The deprotection reaction involves microwave (MW) exposure of diluted reactants in the presence of solid sulphated zirconia (SZ) catalyst that can be easily recovered and reused. The sulphated zirconia was recycled several times without any loss of activity.

## Introduction

Organic transformations are frequently assisted by heterogeneous catalysts which usually provide rate enhancements, yield and/or selectivity improvements, and easier work ups, but the most important general advantage of these methods is their contribution to eco-friendly, “green”, sustainable chemistry, which involves the design of chemical processes with a view to reducing or even eliminating the use and production of hazardous materials. Recent efforts have focused on limiting the use of organic solvents and their replacement with new, less polluting and cost-effective ecologically-friendly processes, a topic of considerable interest to the chemical industry [1,2,3].

Functional group protection and deprotection strategies are essential to target-oriented synthesis in organic chemistry [4,5]. Aldehydes can be protected as acylals (1,1-diacetates), which are stable compounds in basic and neutral media and easily re-converted into parent aldehydes [6]. In general, 1,1-diacetates are prepared from aldehydes by treatment with acetic anhydride using homogeneous catalysts [7,8,9,10,11,12,13,14,15,16,17]. The methods mentioned above are not entirely satisfactory, owing to the problems of corrosion, tedious work-up, environmental pollution and non recoverable catalysts. Such environmental problems have attracted considerable attention towards the development of alternative processes using novel heterogeneous catalysts [18,19,20,21,22,23,24,25,26,27,28,29]. In this context, heterogeneous catalysts play a dramatic role with their easy work-up, separation of the catalyst by filtration, high purity of the products, and the possibility of catalyst recycling. The application of sulphated zirconia as a versatile catalyst in organic transformations was enhanced by the comprehensive review published by Reddy *et al.* [30], and other research groups in subsequent papers [31,32,33]. Following our work on the application of sulphated zirconia as an eco-friendly catalyst [34,35,36,37,38], we proposed its application in protection reactions under solvent free conditions, the deprotection of the protected moieties assisted by microwaves and the synthesis of anhydro-dimers of *o*-hydroxy-benzaldehydes. 

**Figure 1:**
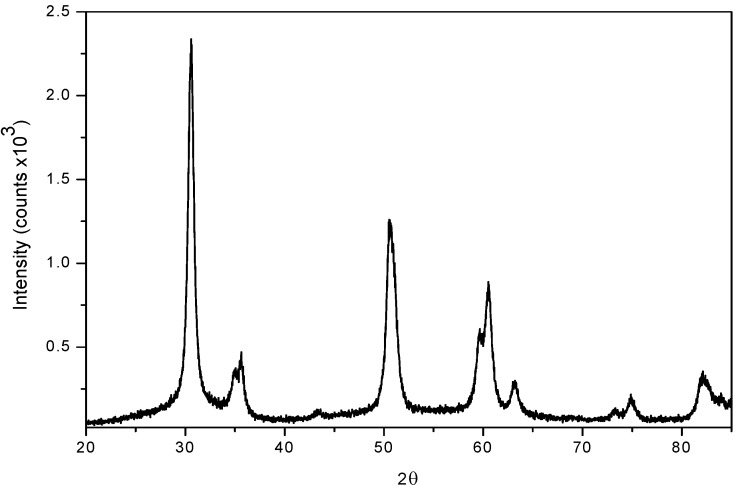
Diffraction pattern of sulphated zirconia.

## Results and Discussion

The X ray powder diffraction (XRD) patterns in the 20–65º 2θ range for sulphated zirconia catalysts showed that after calcination the tetragonal phase is the major form present (ICSD collection code: 066787), as given by reflections in 2θ = 30.18º (with 100 as relative intensity) and the peaks located at 34.616º, 35.283º, 43.002º, 50.214º, 50.770º, 59.291º, 60.187º, 62.724º, 72.894º, 74.617º and 81.768º [35] (Figure 1).

**Figure 2:**
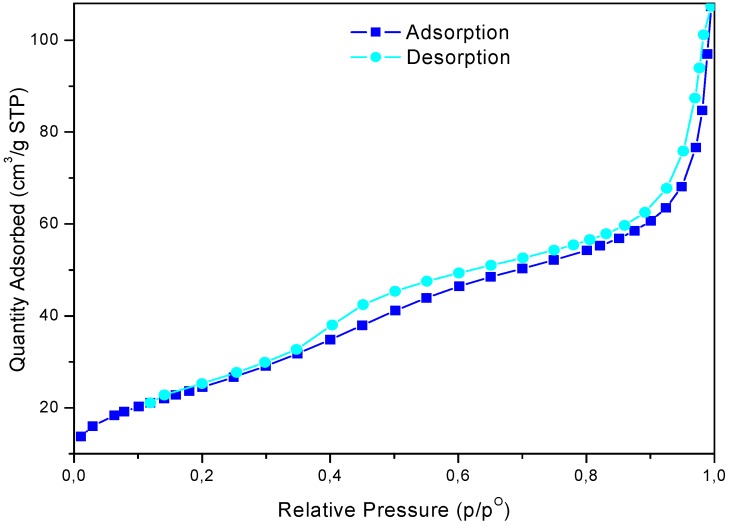
Nitrogen adsorption-desorption plot of sulphated zirconia.

The study of the nitrogen physisorption provides information regarding the textural properties of the catalysts (Figure 2). The adsorption/desorption isotherm relative to calcinated sample belongs to type IV IUPAC classification, with a type H2-H3 hysteresis loop [39]. It is an isotherm with a steep increment of the adsorbed volume at low pressure and a second one at around P/Po = 0.35, corresponding to pore filling and the existence of mesopores [40]. The textural properties of sulphated zirconia are shown in Table 1. The surface area (S_BET_) was calculated by the BET method, the cumulative pore volume (Vp) and the average pore diameter (Dp), were obtained by the BJH data analysis [41].

**Table 1:** Sulphated zirconia surface features.

**Surface area (S_BET_)**	90 m²/g
**Pore volume (Vp)**	0.12 cm³/g
**Pore diameter (Dp)**	52.7 Å

**Figure 3:**
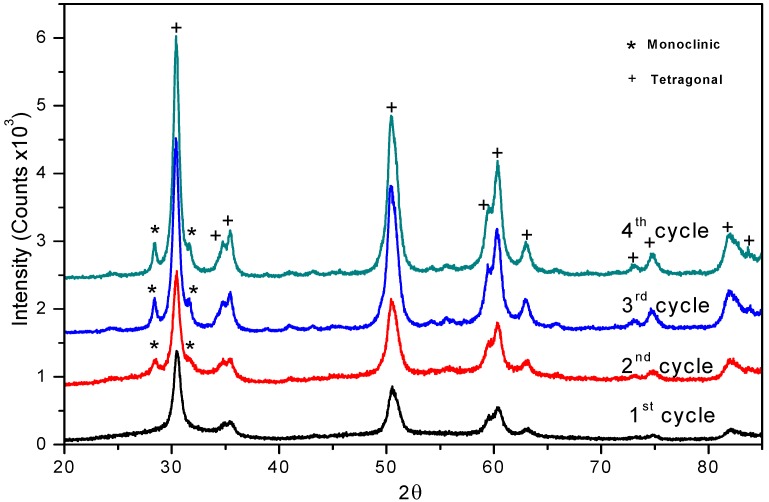
XRD Patterns of reactivated sulphated zirconia.

For testing recyclability, the sulphated zirconia catalyst was filtered after the benzaldehyde protection-deprotection reaction and activated at 500 ºC for 1 hr in air flow; the reactivated sulphated zirconia samples were analyzed by XRD. We observed the presence and increase of the monoclinic phase from the second to the fourth recalcination cycles (2θ = 28.16º and 2θ = 31.44º reflexions), due to the effect of the reactivation process (Figure 3). In a previous work we demonstrated a similar behavior in the synthesis of β-amino alcohols. [37,38]

**Scheme 1:**
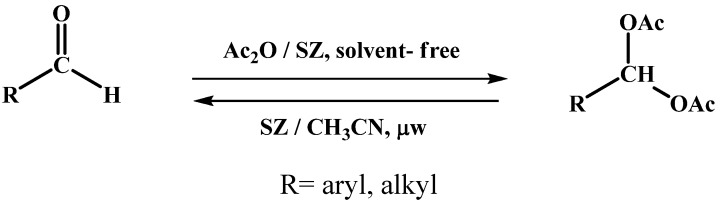
Reaction of protection and deprotection of acylals.

The basic scheme of the acylal protection and deprotection reactions of using sulphated zirconia as acid catalyst is shown in Scheme 1.

Table 2 shows isolated yields using some different aldehydes in the solvent free protection reaction at 0 ºC, in the presence of sulphated zirconia catalyst.

**Table 2:** Aldehydes protection under solvent-free conditions.

Entry	Substrate	Time (hr)	Product	Yield (%)
**1**	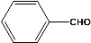	**5.00^a ^**	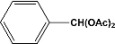	**99^c^**
		**6.00^b^**		**97^d^**
**2**	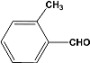	**4.20^a^**	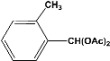	**100^c^**
		**5.00^b^**		**99^d^**
**3**	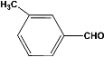	**4.30^a^**	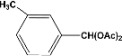	**99^c^**
		**8.00^b^**		**90^d^**
**4**	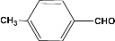	**4.30^a^**	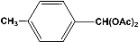	**97^c^**
		**5.00^b^**		**94^d^**
**5**	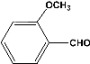	**4.00^a^**	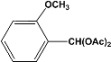	**99^c^**
		**5.00^b^**		**97^d^**
**6**	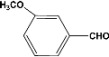	**7.00^a^**	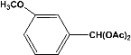	**95^c^**
		**4.30^b^**		**90^d^**
**7**	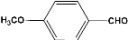	**5.00^a^**	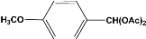	**99^c^**
		**5.00^b^**		**95^d^**
**8**	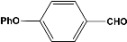	**5.00^a^**	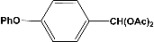	**99^c^**
		**5.00^b^**		**85^d^**
**9**	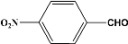	**5.00^a^**	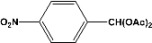	**100^c^**
		**5.00^b^**		**90^d^**
**10**	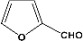	**5.00^a^**	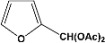	**99^c^**
		**5.00^b^**		**85^d^**
**11**	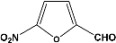	**8.00^a^**	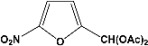	**99^c^**
		**5.00^b^**		**95^d^**

^a ^Reaction conditions: aldehyde (0.83 mmol), acetic anhydride (2.5 mmol), SZ (25 mg) and no solvent at 0 °C; ^b ^Reaction conditions of the published data [35]. ^c ^Yields of solvent free reactions. The products were characterized by IR, ^1^H-NMR and Mass Spectroscopy; ^d^ Yields refer to the published data [35].

Table 3 shows the yield of the 1,1-diacetate deprotections to the corresponding aldehydes using catalytic amounts of sulphated zirconia in CH_3_CN at 60 ºC in a microwave reactor.

**Table 3:** 1,1-Diacetates deprotection reaction to aldehydes.

Entry	Substrate	Time(hr)	Product	Yield (%)
**1**	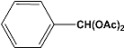	**0.45^a^**	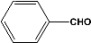	**100^c,d^**
		**6.00^b^**		
**2**	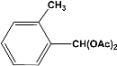	**0.45^a^**	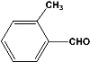	**100^c,d^**
		**6.00^b^**		
**3**	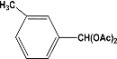	**0.30^a^**	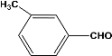	**100^c,d^**
		**6.00^b^**		
**4**	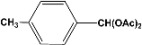	**0.30^a^**	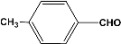	**100^c,d^**
		**6.00^b^**		
**5**	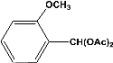	**0.30^a^**	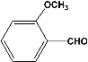	**100^c,d^**
		**6.00^b^**		
**6**	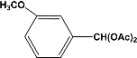	**1.00^a^**	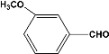	**100^c,d^**
		**6.00^b^**		
**7**	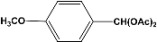	**0.30^a^**	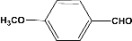	**100^c,d^**
		**6.00^b^**		
**8**	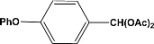	**2.00^a^**	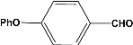	**100^c,d^**
		**6.00^b^**		
**9**	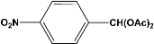	**0.60^a^**	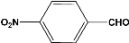	**100^c,d^**
		**6.00^b^**		
**10**	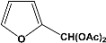	**2.00^a^**	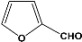	**100^c,d^**
		**6.00^b^**		
**11**	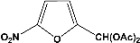	**2.00^a^**	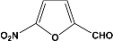	**100^c,d^**
		**6.00^b^**		

^a ^Reaction conditions: A solution of 1,1-diacetates (1 mmol) in HPLC acetonitrile (0.5 mL) and SZ (25 mg) were irradiated at 60 ºC (initial power of 100 W) for 10 min in a self tuning mode CEM lab mate ® microwave synthesizer; ^b ^Reaction conditions of the published data [35]; ^c ^Yields of microwave reactions; ^d^ Yields refer to the published data [35].

When the recycled catalysts were tested in the benzaldehyde protection-deprotection reaction, no significant yield changes were observed (Table 4). 

**Table 4:** Aldehyde protection-deprotection reactions in the presence of reactivated sulphated zirconia.

Cycle No.	% Yield Protection	% Yield Deprotection
1^st^	92	90
2^nd^	90	89
3^rd^	90	89
4^th^	86	87

**Scheme 2:**
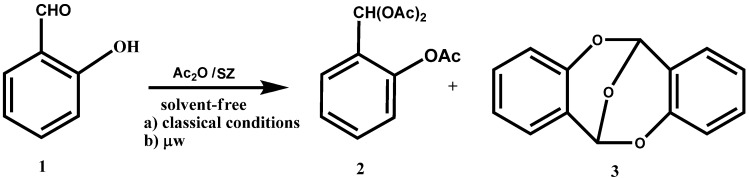
Acylation and autocondensation reaction of **1 **under classic conditions and by microwaves.

We have recently applied the experimental conditions described above to *o*-hydroxybenzaldehyde (**1**)**. **We observed a 12% production of the triacetate **2**, and 88% of the anhydro dimer of *o*-hydroxybenzaldehyde (6,12-epoxy-6H,12H-dibenzo[b,f][1,5]dioxocin, **3**) (Scheme 2).

The classic method of dimerization employs acetic anhydride as the dehydrating agent and solvent using a catalytic amount of sulfuric acid [42], this method provides a 65% of the dimer **3.** Clearly, the advantages with our experimental conditions are the reusability of the catalyst, the easy separation of pure products and carrying out the reaction in conditions of green chemistry.

If the reaction is assisted by microwave radiation at 0 °C, the formation of the dimer of *o*-hydroxy-benzaldehyde, **3**, increases to 93% and the formation of the triacetate, **2** decreases to 7% (Scheme 2).

In our last work, we demonstrated that some aliphatic and aromatic ketones do not form acylals under the described conditions [35]. From this evidence we studied the dimerization reaction of *o*-hydroxybenzaldehyde and *o*-hydroxyacetophenone.

**Scheme 3:**
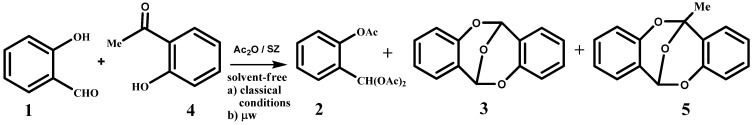
Acylation and condensation reaction of the mixture composed of **1** and **4** under classical conditions and by microwaves.

The dimer **3 **of *o*-hydroxybenzaldehyde has been previously described as the result of an aggressive procedure involving H_2_SO_4_, and the dimer **5 **(6-methyl-6,12-epoxy-6H,12H-dibenzo[b,f][1,5]-dioxocin), is formed in two steps: aggressive dimerization reaction with THF (tetrahydrofuran) at -78 ºC, *n*-butyllithium (*n*-BuLi ) and H_2_SO_4_, followed by a methylation with methyl iodide [43]. Based on the last the last evidence, our aim was the dimerization reaction between *o*-hydroxybenzaldehyde, **1**, and *o*-hydroxyacetophenone, **4 **employing sulphated zirconia as catalyst; we observed the formation of 5% of the corresponding triacetate **2**, 17%yield of the dimer **3 **of *o*-hydroxybenzaldehyde and an 18% yield of dimer **5** (Scheme 3).

Further, when we applied microwave radiation at 0 ºC (initial power 100 W), 5% of product **2** is obtained, 12%of product 3 and 18% of product **5** (Scheme 3).

The importance of these anhydro dimers is that they have four of the six rings present in some natural products, and for this reason, Taylor and co-workers have used them as starting materials in the formation of the preussomerins [43].

**Scheme 4:**
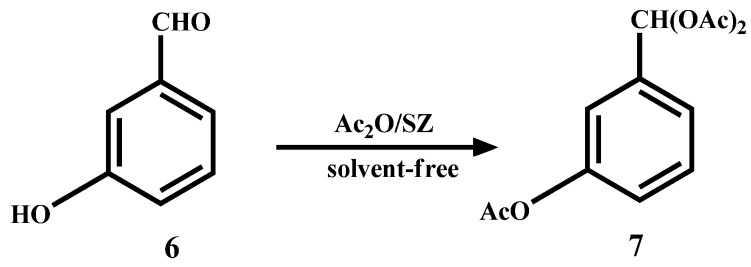
Acylation and acetylation reactions of *m*-hydroxybenzaldehyde.

Furthermore, if *m*-benzaldehyde (**6**) is used as substrate, the formation of the β-hydroxyaldehyde dimerization product is not observed and rather the triacetate **7 **is obtained quantitatively (Scheme 4).

**Scheme 5:**
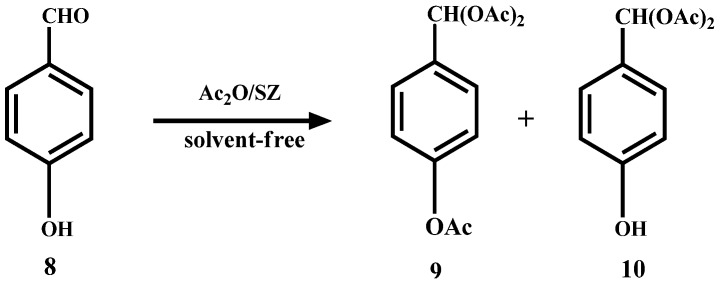
Acylation and acetylation reactions of *p*-hydroxybenzaldehyde.

In the case of *p*-hydroxybenzaldehyde (**8**), it does not form the dimer but, also the triacetal **9** and diacetal **10** are formed in 62% and 18% yield, respectively (Scheme 5).

## Experimental

### General

Powder X ray diffraction (XRD) was performed with a Philips X´Pert Instrument using Cu K-alpha radiation (45 kV, 40 mA). Nitrogen adsorption/desorption isotherms were obtained at -196 ºC on Micrometrics ASAP 2020 equipment. The reaction products were analyzed by GC-FID (Agilent Technologies 6890N) equipped with a HP-5 column with the program 70-180 ºC (20 ºC/min) for 6.50 min then 180-280 ºC (15 ºC/min) for 7 min, injector 250 ºC , detector 280 ºC; the detector was set in the Chemical Ionization mode using methane as reactive gas. Mass spectra were obtained by GC-MS (Agilent Technologies 6890N, Detector 5973) using methane chemical ionization. ^1^H-NMR and ^13^C-NMR spectra was measured at 500 MHz and 125 MHz, respectively, on a Bruker Avance DMX-500 spectrometer using CDCl3 as solvent and tetramethylsilane as internal standard. Infrared (IR) spectra were recorded on a Bruker Vector 33 ATR spectrometer. All aldehydes, reagents and solvents were provided by Aldrich and all products were identified by IR, ^1^H-NMR, ^13^C-NMR, mass spectra and by comparison of their corresponding melting points. 

### Sulphated zirconia synthesis

Zirconium *n*-propoxide (20 mL, 70% *n*-propanol) was mixed with 2-propanol (30 mL) and stirred with a magnetic bar. Then, an acid solution (1 mL 98% sulfuric acid in 3.2 mL of distilled water) was added dropwise in order to cause the hydrolysis and gelation of the zirconium *n*-propoxide. The solid was filtered and dried at 80 ºC until complete alcohol evaporation, then calcinated in air at 600 ºC for 6 h.

### Procedure for the conversion of aldehydes to geminal diacetates under solvent free conditions

A mixture of aldehyde (0.83 mmol), acetic anhydride (2.50 mmol), and sulphated zirconia (25 mg) were placed in a screw-cap vial at 0 ºC. The suspension was stirred for the appropriate time (see Table 1). After completion of the reaction, the catalyst was recovered by filtration and the filtrate was extracted with EtOAc (3x10 mL) and water (2 × 10 mL). The organic extracts were combined and washed with 10% NaHCO_3_ and dried over anhydrous Na_2_SO_4_. The solvent was evaporated under reduced pressure and the residue was purified on silica gel using light petroleum-ether as eluent to give the pure product 1,1-diacetates. All products were identified via GC-FID mass spectra and analyzed GC-MS, ^1^H-NMR, ^13^C-NMR.

### Procedure for the deprotection of 1,1 diacetates to aldehydes using Microwave synthesizer

A solution of 1,1-diacetate (1 mmol) in HPLC-grade acetonitrile (0.5 mL) and sulphated zirconia (25 mg) was introduced into a pressurized reaction tube (10 mL) equipped with a magnetic stirrer and the mixture was irradiated at 60 ºC (initial power of 100 W) for 10 min in a self tuning mode CEM Labmate ® microwave synthesizer. After the reaction, the catalyst was recovered by filtration and the filtrate was extracted with EtOAc and washed with water. The organic extracts were combined and washed with 10% NaHCO_3_ and dried over anhydrous Na_2_SO_4_. Then was filtered and evaporated under reduced pressure to give the corresponding aldehyde.

### Procedure for the formation of o-hydroxybenzaldehyde dimer (6,12-epoxydibenzo-6H,12H-dibenzo[b,f][1,5] dioxocin, ***3***) using the conventional thermal method

A mixture of *o-*hydroxybenzaldehyde (0.1738 mL, 1.66 mmol), acetic anhydride (0.1181 mL, 1.25 mmol) and sulphated zirconia (50 mg) were stirred for 3 h. under solvent free conditions. After completion of the reaction, the catalyst was recovered by filtration and the filtrate was extracted with EtOAc (3 × 10 mL) and washed with water (2 × 10 mL). The organic extracts were combined and washed with 10% NaHCO_3_ and dried over anhydrous Na_2_SO_4_. The solvent was evaporated under reduced pressure and the residue was chromatographied on silica gel (light petroleum-ether as eluent) to give the corresponding dimer **3 **as a white solid in 80% yield; m.p. 127-130 ºC (lit. [44] m.p. 130 ºC); GC-MSD for C_14_H_10_O_3_ (m.w.: 226g/mol): [M+1]^+ ^= 227, [M+29]^+ ^= 255, [M+41]^+ ^= 267; IR (cm^-1^):1590, 1455, 1069, 1611, 1217. ^1^H-NMR: δ (ppm) 7.28 (dd, 2H, *J1* = 1.6, *J2* = 7.7 Hz), 7.21-7.26 (m, 2H), 6.95 (ddd, 2H, *J1* = 1.1, *J2* = 7.5, *J3* = 8.6 Hz), 6.87 (d, 2H, *J* = 8.2 Hz), 6.35 (s, 2H); ^13^C-NMR: δ (ppm) 150.50, 130.94, 127.47, 121.51, 119.98, 116.59, 90.13. (lit. [45] ^13^C-NMR (125 MHz, CDCl3): δ (ppm) 150.55, 130.89, 127.42, 121.46, 120.00, 116.58, 90.14).

### Procedure for the formation of o-hydroxybenzaldehyde dimer (6,12-epoxydibenzo-6H,12H-dibenzo[b,f][1,5] dioxocin, ***3***) using microwave irradiation

A mixture of *o*-hydroxybenzaldehyde (0.1738 mL, 1.66 mmol), acetic anhydride (0.1181 mL, 1.25 mmol) and sulphated zirconia (50 mg) were introduced into a pressurized tube (10 mL) equipped with a magnetic stirrer, which was irradiated in solvent free conditions to 0 ºC (initial power of 100 W) for 30 min. After reaction, the catalyst was recovered by filtration and the filtrate was treated as described in the conventional thermal method described above for dimer **3**.

### Procedure for the preparation of the dimer 6-methyl-6,12-epoxy-6H,12H-dibenzo[b,f][1,5]dioxocin *(**5**)* using the conventional thermal method

A mixture of *o*-hydroxybenzaldehyde (0.0869 mL, 0.83 mmol), *o*-hydroxyacetophenone (0.2260 mL, 1.66 mmol), acetic anhydride (0.11801 mL, 1.25 mmol) and sulphated zirconia (50 mg) was magnetically stirred in solvent-free conditions at 0 ºC for 6 h. After completion of the reaction, the catalyst was recovered by filtration and the filtrate was extracted with EtOAc (3x10 mL) and washed with water (2x10 mL). The organic layer was washed with 10% NaHCO_3_ and dried over anhydrous Na_2_SO_4_. The solvent was evaporated under reduced pressure and the residue was purified by silica gel chromatography using 100% light petroleum-ether as eluent to give the corresponding dimer **5** in 15% as a white solid with a m.p. 80-83 °C, GC-MSD for C_15_H_12_O_3_ (m.w.: 240 g/mol): [M+1]^ + ^= 241, [M+29]^+ ^= 269, [M+41]^+ ^= 281; IR (cm^-1^): 1383, 1590, 1455, 1069, 1611, 1217; ^1^H-NMR: δ (ppm) 7.33 (ddd, 1H, *J1* = 0.4, *J2* = 1.6, *J3* = 7.8 Hz), 7.27 (dtd, 1H, *J1* = 0.5, *J2* = 1.7, *J3* = 7.7 Hz), 7.14-7.19 (m, 2H), 6.91 (dd, 1H, *J1* = 1.2, *J2* = 7.4, Hz), 6.88 (dd, 1H, *J1* = 1.2, *J2* = 7.4, Hz), 6.83 (ddd, 1H, *J1* = 0.4, *J2* = 1.2, *J3* = 8.2 Hz), 6.79-6.81 (m, 1H), 6.33 (s,1H), 2.01 (s, 3H);^13^C-NMR: δ (ppm) 151.98, 150.23, 130.32, 130.71, 127.26, 126.01, 123.42, 121.62, 121.22, 119.55, 116.76, 116.29, 95.45, 90.97, 25.05.

### Procedure for the preparation of the dimer 6-methyl-6,12-epoxy-6H,12H-dibenzo[b,f][1,5]dioxocin *(**5**)* using microwave irradiation

In a glass tube for microwave reactions equipped with a magnetic stirrer were introduced a suspension of sulphated zirconia (50 mg), *o*-hydroxybenzaldehyde (0.0869 mL, 0.83 mmol), *o*-hydroxyacetophenone (0.2260 mL, 1.66 mmol) and acetic anhydride (0.1181 mL, 1.25 mmol) in under solvent free conditions at 0 ºC and the mixture was irradiated (initial power of 100 W) for 1 h. After the reaction time, the catalyst was recovered by filtration and the filtrate was treated as described in the conventional thermal method described above for dimer **5**.

## Conclusions

We have demonstrated the high activity of sulphated zirconia in the solvent-free formation of glycals (1,1-diacetates or acylals), and in the corresponding microwave assisted deprotection reactions. The catalyst can be recycled several times without significant loss of activity, it is highly stable, chemoselective, and environmentally friendly, which could open the possibility for an environmentally benign approach for the synthesis of acylals and triacetals under mild reaction conditions. 

In case of *o*-hidroxybenzaldehyde, we observed the formation of the corresponding anhydro dimer, as well as the formation of an acetylation product. For *m*-hydroxybenzadehyde and *p*-hydroxy-benzaldehyde, aside from the acylation reaction, acetylation also occurs. On the other hand, when we used *o*-hydroacetophenone with the *o*-hydroxybenzaldehyde, we observed the formation of the corresponding anhydro dimer methylated on the six position.
